# Efficacy and safety of transarterial chemoembolization combining sorafenib with or without immune checkpoint inhibitors in previously treated patients with advanced hepatocellular carcinoma: A propensity score matching analysis

**DOI:** 10.3389/fonc.2022.914385

**Published:** 2022-09-13

**Authors:** Xue-Gang Yang, Yan-Yuan Sun, Hai-Qing Wang, De-Shan Li, Guo-Hui Xu, Xiao-Qi Huang

**Affiliations:** ^1^ Department of Interventional Radiology, Sichuan Cancer Hospital and Institute, Sichuan Cancer Center, Chengdu, China; ^2^ Huaxi MR Research Center (HMRRC), Functional and molecular imaging Key Laboratory of Sichuan Province, Department of Radiology, West China Hospital, Sichuan University, Chengdu, China; ^3^ Department of Hepato-Biliary-Pancreatic Surgery, Sichuan Cancer Hospital and Institute, Sichuan Cancer Center, Chengdu, China

**Keywords:** hepatocellular carcinoma, transarterial chemoembolization, sorafenib, immune checkpoint inhibitor, PD-1 inhibitor, combined therapy

## Abstract

**Purpose:**

To compare the efficacy and safety of transarterial chemoembolization (TACE) plus sorafenib and immune checkpoint inhibitors (T+S+ICIs) and TACE plus sorafenib (T+S) when treating patients with advanced hepatocellular carcinoma (HCC) who have previously received locoregional treatment.

**Materials and methods:**

A retrospective analysis was performed on the patients with Barcelona Clinic Liver Cancer (BCLC) stage C HCC from May 2019 to December 2020. These patients were treated with locoregional therapy and showed radiographic progression after the treatment. Patients received either T+S+ICIs or T+S. The outcomes, including disease control rate (DCR), progression-free survival (PFS), overall survival (OS), and safety, were compared. The propensity score matching (PSM) methodology was used to reduce the influence of confounding factors on the outcomes.

**Results:**

Forty-three patients were included in the T+S group and 33 in the T+S+ICI group. After PSM (n = 29 in each group), patients who received T+S+ICIs had a higher DCR (82.8% vs. 58.6%, p = 0.043), longer median PFS (6.9 vs. 3.8 months, p = 0.003), and longer median OS (12.3 vs. 6.3 months, p = 0.008) than those who underwent T+S. Eastern Cooperative Oncology Group performance status was an independent predictor of PFS, and age was an independent predictor of OS. The incidence of treatment-related adverse events in T+S+ICIs was well controlled.

**Conclusions:**

Compared with TACE combined with sorafenib, TACE combined with sorafenib plus ICIs is a potentially safe and effective treatment regimen for patients with advanced HCC who previously received locoregional treatment.

## Introduction

Clinical practice guidelines have recommended transarterial chemoembolization (TACE) for intermediate-stage HCC treatment (1, 2). In addition, the application scope of TACE has been expanded from Barcelona Clinic Liver Cancer (BCLC) stage A to stage C according to the Chinese guidelines for the diagnosis and treatment of HCC (3). However, TACE may increase tumor hypoxia, leading to the upregulation of vascular endothelial growth factor (VEGF) and platelet-derived growth factor (PDGF), promotion of tumor angiogenesis (4), and tumor recurrence or metastasis.

Sorafenib is a protein kinase inhibitor that hampers the activities of protein kinases, including VEGF, RAF, and PDGF, thereby exerting both antiangiogenic and direct antitumor effects. Some studies have shown that sorafenib combined with TACE treatment prolongs the progression-free survival (PFS) (5) and overall survival (OS) of patients with intermediate-advanced HCC (6). However, data from two phase II/III randomized controlled trials (RCTs), including TACE 2 trial (7) and SPACE trial (8), failed to demonstrate any clinical benefit of sorafenib combined with TACE. Thus, effective systemic therapies combined with TACE are urgently needed to improve the prognosis of patients.

Immune checkpoint inhibitors (ICIs) have shown promising clinical outcomes, and pembrolizumab and nivolumab have been approved by the US Food and Drug Administration (FDA) as a second-line systemic treatment for HCC based on phase I/II study results (9, 10). Atezolizumab combined with bevacizumab has shown the better PFS and OS than sorafenib in unresectable HCC (11).

Since TACE has local anticancer effects, it may promote antitumor immunity but inevitably induce post-TACE angiogenesis (12, 13), and sorafenib can promote “tumor vascular normalization” to alleviate hypoxia and therefore enhance the efficacy of TACE and immunotherapy. ICIs may restore the immune-supportive tumor microenvironment (TME) through inhibiting immune checkpoints. Studies have suggested the potential synergistic antitumor immunomodulatory effect when combining ICIs with other antitumor approaches to stimulate the immune system or directly kill tumor cells (14–16). In this study, we hypothesized that the comprehensive therapy of TACE plus sorafenib and ICIs might improve the treatment outcomes of patients with advanced HCC. Therefore, we compared the efficacy and safety of the TACE+sorafenib+ICI (T+S+ICI) regimen with those of the TACE+sorafenib (T+S) regimen in treating patients with BCLC stage C HCC who have previously received locoregional treatment.

## Materials and methods

### Study design and patient selection

This was a retrospective study that was conducted in accordance with the principles of the Declaration of Helsinki. Ethics approval was obtained from the ethical review committee of Sichuan Cancer Hospital. Informed consent was obtained from available patients and was waived in the case of deceased or otherwise unattainable patients.

Patients diagnosed with BCLC C stage HCC from 1 May 2019 to 31 December 2020, based on the HCC guidelines of the European Association for the Study of Liver, were eligible for enrollment (2). Portal vein tumor thrombus (PVTT) was categorized into four types according to the classification criteria proposed by previous authors (17). The inclusion criteria included the following: 1) patients aged between 18 and 80 years; 2) patients who had the Eastern Cooperative Oncology Group performance status (ECOG PS) of ≤2; 3) patients who had the Child–Pugh class A or B; and 4) HCC patients treated with locoregional therapy and radiographic progression seen after treatment. The exclusion criteria were as follows: 1) patients who received TACE combined with sorafenib or TACE combined with sorafenib plus ICIs as the first-line therapy; 2) patients with other malignancies; 3) patients with hepatic encephalopathy, severe ascites, esophageal or gastric fundal variceal bleeding, or other serious medical comorbidities; 4) patients with coagulation disorders; 5) patients who received ICI treatment before TACE; and 6) patients with incomplete treatment or follow-up data.

### TACE procedure

The procedure was performed with the guidance of digital subtraction angiography (DSA). Hepatic artery angiography was performed with a Yashiro-type or 5-F RH catheter (Terumo) to assess the location, number, size, and blood supply of target tumors. Subsequently, a microcatheter (Progreat; Terumo, Ann Arbor, MI, USA) was inserted into the feeding artery of tumors. Intra-arterial administration consisted of 40–60 mg of epirubicin (Pharmorubicin; Pfizer, Wuxi, China) mixed with 5–20 ml of lipiodol (Jiangsu Hengrui Medicine Co., Ltd., Jiangsu, China). Embolization was stopped following stasis of the contrast agent flow. When needed, further embolization was performed with Embosphere (100–300 μm) to achieve stasis.

### Sorafenib and ICI administration

Administration of sorafenib and ICIs was initiated within 1–2 weeks after TACE therapy based on the liver condition (requiring aspartate aminotransferase (AST) level <40 U/l). Sorafenib at a dose of 400 mg was orally administered twice a day, and it was discontinued for 2 days before and after each TACE treatment session (5). Intravenous administration of 200 mg camrelizumab (Jiangsu Hengrui Medicine Co., Ltd., Jiangsu, China) or 200 mg sintilimab (Innovent Biologics, Suzhou, China) was conducted every 3 weeks. The interruption and discontinuation of drug administration depended on the presence and severity of toxic side effects according to the drug directions. Once ICI-related serious adverse events (SAEs) occurred, ICIs were discontinued, and those patients were kept in the T+S+ICI group.

### Follow-up

After the first TACE, the standard-of-care clinical and radiological follow-up was scheduled at 4–6 weeks and every 3 months thereafter. The follow-up results (CT or MR images and laboratory tests) were evaluated by our multidisciplinary team (MDT) to determine the status of tumor lesions (tumor progression or not). All patients were followed up till 31 August 2021.

### TACE retreatment

TACE retreatment was performed only on demand, after MDT discussion, depending upon the extension of the residual or recurrent viable tumor and patients’ clinical conditions. During follow-up, the treatment of T+S+ICIs or T+S was discontinued in case of intolerable toxicity, progressive disease (PD), or change of treatment plan. The choice of the subsequent treatment, such as second-line targeted agent, ICIs (for the patients treated with T+S), radiotherapy, or best supportive care, was determined according to the results of discussion by our MDT and the patients’ request.

### Treatment evaluation

Tumor responses were evaluated by two diagnostic radiologists with more than 10 years of experience according to the modified Response Evaluation Criteria in Solid Tumors (mRECIST). Objective response rate (ORR) was defined as the proportion of patients achieving complete response (CR) or partial response (PR). Disease control rate (DCR) was defined as the rate of objective response plus stable disease (SD). All objective responses were confirmed at least 4 weeks after the first observation of all patients.

PFS was defined as the time interval between the TACE procedure and the time of disease progression due to any cause. OS was defined as the period from the TACE procedure to the time of death or the last date of follow-up. Adverse events (AEs) were recorded and assessed based on the Common Terminology Criteria for Adverse Events Version 5.0.

### Statistical analysis

Statistical analysis was performed using SPSS 25.0 (IBM). The propensity score model enrolled the following variables: age, sex, ECOG PS, hepatitis B surface antigen level, AFP, Child–Pugh class, and intrahepatic major tumor size. The 1:1 nearest-neighbor method was used to deduce the matched pairs between the two groups, with a caliper width of 0.03 of the standard deviation of the logit of the propensity score. Before and after propensity score matching (PSM), the quantitative data were expressed as frequency, mean ± standard deviation (SD), or median with a 95% confidence interval (CI). To determine the significant differences between the two groups, continuity correction and independent-samples t-test, chi-square test, or Fisher’s exact test were used. Survival curves of PFS and OS were analyzed by the Kaplan–Meier method using the log-rank test. The Cox proportional hazard model was used for univariate and multivariate analyses to determine the prognostic factors. All statistically significant (p < 0.15) factors identified by the univariate analysis were entered into a Cox proportion hazards regression model to identify the independent predictors. All statistical analyses were based on the two-tailed hypothesis tests with a significance level of p < 0.05.

## Results

### Patient characteristics

**Figure 1:**
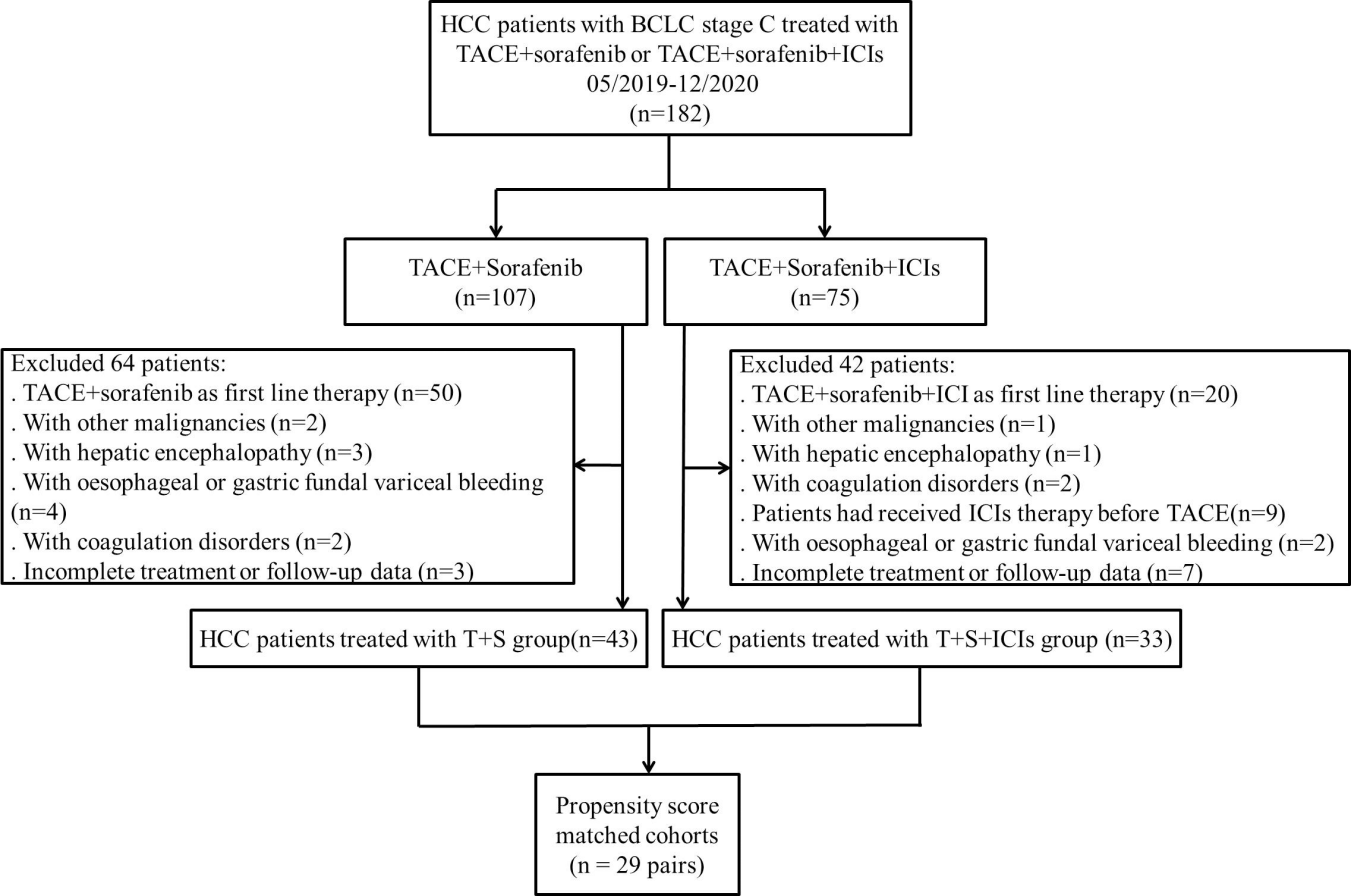
Flowchart shows the patients’ selection process. BCLC, Barcelona Clinic Liver Cancer; HCC, hepatocellular carcinoma; TACE, transarterial chemoembolization; ICIs, immune checkpoint inhibitors; T+S, transarterial chemoembolization+sorafenib; T+S+ICIs, transarterial chemoembolization+sorafenib+immune checkpoint inhibitors.

Seventy-six patients with BCLC C stage HCC were included in this study. The average tumor size was 9.6 ± 4.8 cm. There were 43 patients in the T+S group and 33 patients in the T+S+ICI group (**Figure 1**). Nineteen patients received camrelizumab, and 14 patients received sintilimab in the T+S+ICI group.

Following PSM, 58 patients were analyzed (29 patients in the T+S group and 29 patients in the T+S+ICI group) (**Figure 1**). The baseline characteristics before and after PSM of the two groups were similar (p > 0.05) (**Table 1**).

**Table 1:** Patient demographics and baseline characteristics before and after propensity score matching.

Characteristics	Before PSM			After PSM		
	T+S (n = 43)	T+S+ICIs (n = 33)	p value	T+S (n = 29)	T+S+ICIs (n = 29)	p value
Age (years) <50 ≥50	50.9 ± 11.5 16 (37.2) 27 (62.8)	54.6 ± 9.7 16 (48.5) 17 (51.5)	0.324	51.3 ± 11.2 13 (44.8) 16 (55.2)	53.7 ± 10.2 14 (48.3) 15 (51.7)	0.792
Sex			0.434			>0.999
Men	39 (90.7)	28 (84.8)		26 (89.7)	26 (89.7)	
Women	4 (9.3)	5 (15.2)		3 (10.3)	3 (10.3)	
ECOG PS			0.987			0.945
0 1 2	9 (20.9) 31 (72.1) 3 (7.0)	7 (21.2) 24 (72.7) 2 (6.1)		6 (20.7) 21 (72.4) 2 (6.9)	5 (17.2) 22 (75.9) 2 (6.9)	
HBV			0.827			>0.999
Positive	36 (83.7)	27 (81.8)		24 (82.8)	24 (82.8)	
Negative	7 (16.3)	6 (18.2)		5 (17.2)	5 (17.2)	
Cirrhosis			0.610			0.773
Yes	31 (72.1)	22 (66.7)		21 (72.4)	20 (69.0)	
No	12 (27.9)	11 (33.3)		8 (27.6)	9 (31.0)	
Child–Pugh			0.109			0.773
A	25 (58.1)	25 (75.8)		20 (69.0)	21 (72.4)	
B	18 (41.9)	8 (24.2)		9 (31.0)	8 (27.6)	
AFP (ng/mL)			0.339			>0.999
<400	23 (53.5)	14 (42.4)		12 (41.4)	12 (41.4)	
≥400	20 (46.5)	19 (57.6)		17 (58.6)	17 (58.6)	
AST (U/L) ALT (U/L) Albumin (g/L)	75.3 ± 62.3 37.1 ± 24.3 34.0 ± 5.0	69.9 ± 60.9 44.3 ± 35.7 35.8 ± 5.4	0.706 0.299 0.130	83.2 ± 70.6 39.1 ± 26.9 34.3 ± 5.2	74 ± 63.9 45.4 ± 37.8 35.2 ± 4.6	0.854 0.616 0.625
Tumor size (cm)			0.878			0.599
<10	24 (55.8)	19 (57.6)		13 (44.8)	15 (51.7)	
≥10	19 (44.2)	14 (42.4)		16 (55.2)	14 (48.3)	
Vascular invasion	31 (72.1)	27 (81.8)	0.323	21 (72.4)	24 (82.8)	0.345
EHS	27 (62.8)	17 (51.5)	0.324	17 (58.6)	16 (55.2)	0.791
Type of PVTT I+II III	16 (37.2) 14 (32.6)	17 (51.5) 8 (24.2)	0.269	10 (34.5) 11 (37.9)	16 (55.2) 8 (27.6)	0.197
Number of TACE			0.054			0.146
1 2 3	25 (58.1) 11 (25.5) 7 (16.3)	10 (30.3) 14 (42.4) 9 (27.3)		15 (51.7) 10 (34.5) 4 (13.8)	8 (27.6) 13 (44.8) 8 (27.6)	
Prior therapy			0.687			0.803
DEB-TACE/cTACE DEB-TACE/cTACE+RFA Surgery+cTACE/RFA	29 (67.4) 7 (16.3) 7 (16.3)	20 (60.6) 8 (24.2.) 5 (15.2)		20 (69.0) 5 (17.2) 4 (13.8)	18 (62.1) 7 (24.1) 4 (13.8)	

Data were presented as n (%) or mean ± standard deviation. PSM, propensity score matching; T+S, transarterial chemoembolization+sorafenib; T+S+ICIs, transarterial chemoembolization+sorafenib+immune checkpoint inhibitors; ECOG PS, Eastern Cooperative Oncology Group performance status; HBV, hepatitis B virus; AFP, alpha-fetoprotein; AST, aspartate aminotransferase; ALT, alanine transaminase; EHS, extrahepatic spread; PVTT, portal vein tumor thrombus; type I, tumor thrombi involving segmental branches of portal vein or above; type II, tumor thrombi involving right/left portal vein; type III, tumor thrombi involving the main portal vein; DEB-TACE, drug-eluting bead transarterial chemoembolization; cTACE, conventional transarterial chemoembolization; RFA, radiofrequency ablation.

### Treatment outcomes

#### Tumor response evaluation

The DCR was maintained higher for patients in the T+S+ICI group than for those in the T+S group before (84.8% vs. 55.8%, p = 0.007) and after (82.8% vs. 58.6%, p = 0.043) PSM (**Table 2**). The ORR was higher for patients in the T+S+ICI group than for those in the T+S group before (60.6% vs. 27.9%, p = 0.004) PSM. However, there was no difference in ORR (58.6%% vs. 34.5%, p = 0.065) after PSM (**Table 2**).

**Table 2:** Summary of response rates before and after propensity score matching.

Best overall response, n (%)	Before PSM			After PSM		
	T+S (n = 43)	T+S+ICIs (n = 33)	p value	T+S (n = 29)	T+S+ICIs (n = 29)	p value
Complete response	0 (0)	0 (0)	> 0.999	0 (0)	0 (0)	> 0.999
Partial response	12 (27.9)	20 (60.6)	0.004	10 (34.5)	17 (58.6)	0.065
Stable disease	12 (27.9)	8 (24.2)	0.719	7 (24.1)	7 (24.1)	> 0.999
Progressive disease	19 (44.2)	5 (15.2)	0.007	12 (41.4)	5 (17.2)	0.043
Objective response rate Disease control rate	12 (27.9) 24 (55.8)	20 (60.6) 28 (84.8)	0.004 0.007	10 (34.5) 17 (58.6)	17 (58.6) 24 (82.8)	0.065 0.043

Data are numbers of patients, with percentages in parentheses. PSM, propensity score matching; T+S, transarterial chemoembolization+sorafenib; T+S+ICIs, transarterial chemoembolization+sorafenib+immune checkpoint inhibitors.

### Survival analysis

**Figure 2:**
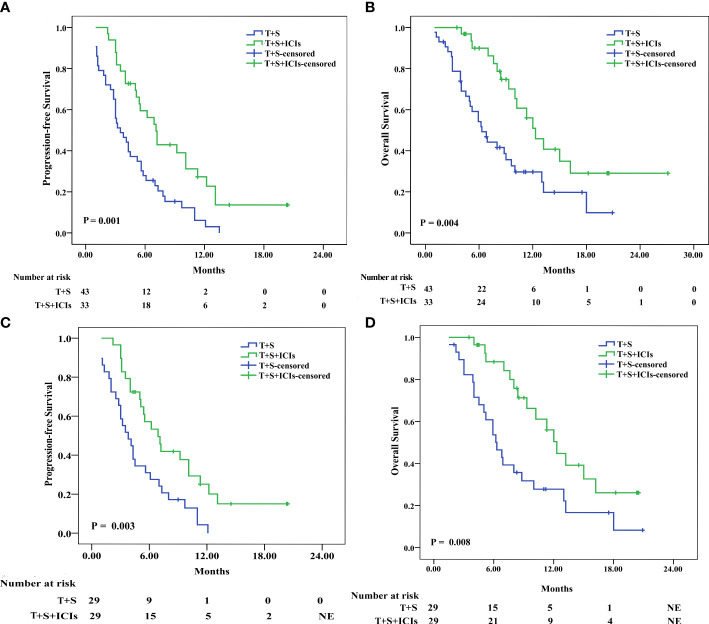
Kaplan–Meier analyses of progression-free survival and overall survival before **(A, B)** and after **(C, D)** propensity score matching in patients treated with T+S or T+S+ICIs. T+S, transarterial chemoembolization+sorafenib; T+S+ICIs, transarterial chemoembolization+sorafenib+immune checkpoint inhibitors.

Before PSM, the median PFS was 7.1 months (95% CI 5.773–8.427) in the T+S+ICI group and 3.5 months (95% CI 2.087–4.913) in the T+S group (p = 0.001) (**Figure 2A**), and the median OS was 12.3 months (95% CI 9.719–14.881) in the T+S+ICI group and 6.3 months (95% CI 4.559–8.041) in the T+S group (p = 0.004) (**Figure 2B**).

After PSM, the median PFS was 6.9 months (95% CI 4.805–8.995) in the T+S+ICI group and 3.8 months (95% CI 2.218–5.383) in the T+S group (p = 0.003) (**Figure 2C**), and the median OS was 12.3 months (95% CI 10.36–14.24) in the T+S+ICI group and 6.3 months (95% CI 4.647–7.953) (p = 0.008) (**Figure 2D**) in the T+S group.

### Subgroup analysis

#### Subgroup analyses of patients in the two groups before PSM

**Figure 3:**
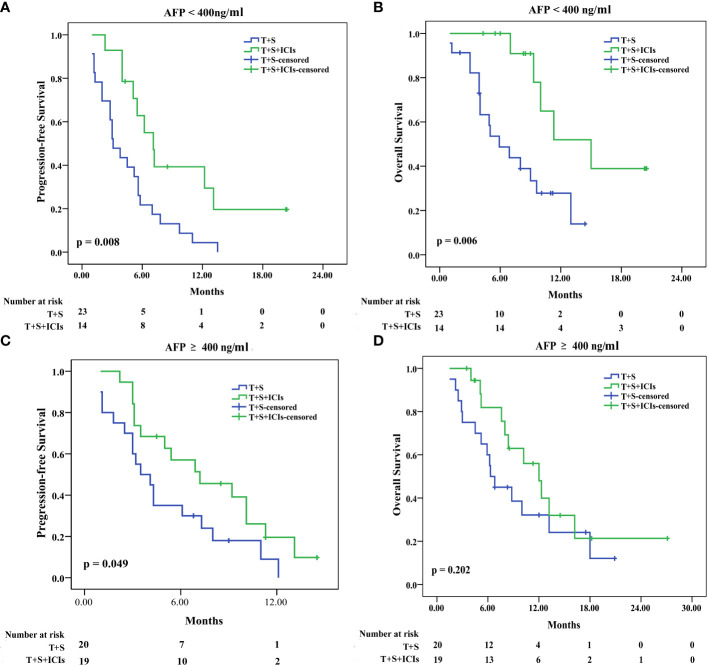
Subgroup analysis for progression-free survival and overall survival stratified by AFP level <400 ng/ml **(A, B)** and ≥400 ng/ml **(C, D)**. T+S, transarterial chemoembolization+sorafenib; T+S+ICIs, transarterial chemoembolization+sorafenib+immune checkpoint inhibitors.

In patients with AFP of <400 ng/ml, the median PFS was 7.2 months (95% CI: 5.374–8.826) in the T+S+ICI group and 3.5 months (95% CI 1.926–4.274) in the T+S group (p = 0.008) (**Figure 3A**); the median OS was 15 months (95% CI: 8.433–21.567) in the T+S+ICI group and 6.3 months (95% CI 2.979–8.821) in the T+S group (p = 0.006) (**Figure 3B**). In patients with AFP of ≥400 ng/ml, the median PFS was 7.1 months (95% CI 2.331–12.069) in the T+S+ICI group and 3.1 months (95% CI 1.528–5.472) in the T+S group (p = 0.049) (**Figure 3C**); the median OS was 12 months (95% CI: 8.634–15.366) in the T+S+ICI group and 5.9 months (95% CI 4.985–7.615) in the T+S group (p = 0.202) (**Figure 3D**).

**Figure 4:**
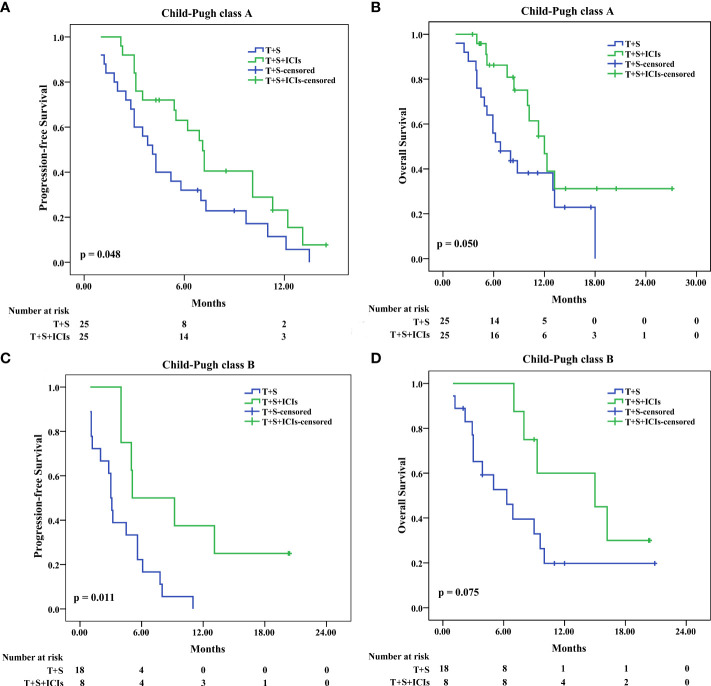
Subgroup analysis for progression-free survival and overall survival stratified by Child–Pugh class A **(A, B)** and B **(C, D)**. T+S, transarterial chemoembolization+sorafenib; T+S+ICIs, transarterial chemoembolization+sorafenib+immune checkpoint inhibitors.

In patients with Child–Pugh class A, the median PFS was 7.1 months (95% CI: 5.970–8.230) in the T+S+ICI group and 4.1 months (95% CI 3.121–5.079) in the T+S group (p = 0.048) (**Figure 4A**); the median OS was 15 months (95% CI 0.979–29.021) in the T+S+ICI group and 6.8 months (95% CI 3.474–10.126) in the T+S group (p = 0.05) (**Figure 4B**). In patients with Child–Pugh class B, the median PFS was 5.1 months (95% CI 0.000–10.921) in the T+S+ICI group and 3 months (95% CI 2.584–3.416) in the T+S group (p = 0.011) (**Figure 4C**); the median OS was 12.0 months (95% CI 9.649–14.351) in the T+S+ICI group and 6.3 months (95% CI 2.618–9.982) in the T+S group (p = 0.075) (**Figure 4D**).

**Figure 5:**
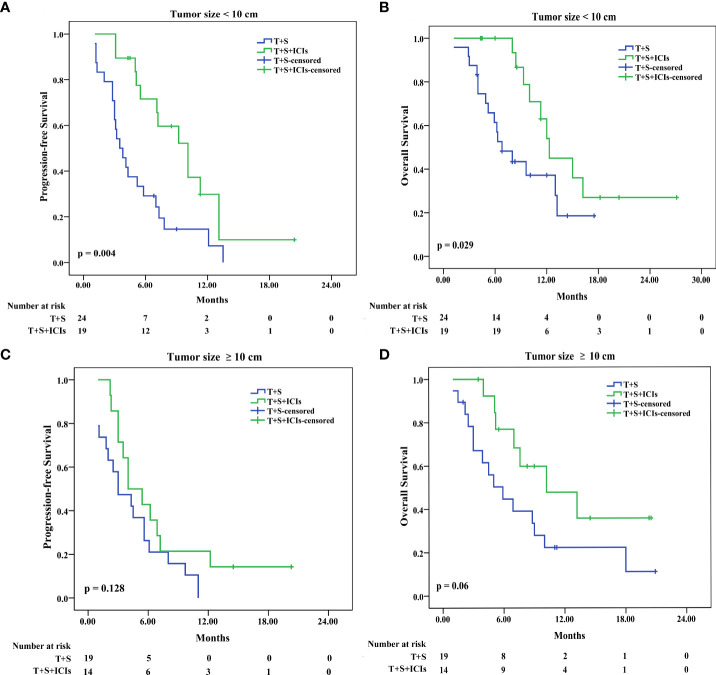
Subgroup analysis for progression-free survival and overall survival stratified by tumor size <10 cm **(A, B)** and ≥10 cm **(C, D)**. T+S, transarterial chemoembolization+sorafenib; T+S+ICIs, transarterial chemoembolization+sorafenib+immune checkpoint inhibitors.

In patients with tumor size of <10 cm, the median PFS was 10.1 months (95% CI 6.894–13.306) in the T+S+ICI group and 3.5 months (95% CI 2.300–4.7) in the T+S group (p = 0.004) (**Figure 5A**); the median OS was 12.3 months (95% CI 8.449–16.151) in the T+S+ICI group and 6.8 months (95% CI 4.097–9.503) in the T+S group (p = 0.029) (**Figure 5B**). In patients with tumor size of ≥10 cm, the median PFS was 4 months (95% CI 1.678–6.322) in the T+S+ICI group and 3 months (95% CI 0.441–5.559) in the T+S group (p=0.128) (**Figure 5C**); the median OS was 10.2 months (95% CI 3.093–17.307) in the T+S+ICI group and 5.9 months (95% CI 3.023–8.777) in the T+S group (p = 0.06) (**Figure 5D**).

**Figure 6:**
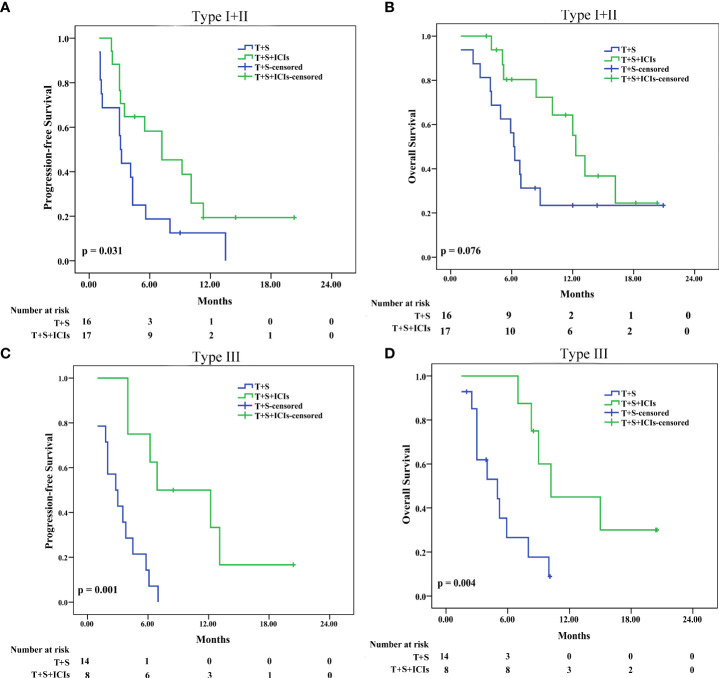
Subgroup analysis for progression-free survival and overall survival stratified by type I+II PVTT **(A, B)** and type III PVTT **(C, D)**. T+S, transarterial chemoembolization+sorafenib; T+S+ICIs, transarterial chemoembolization+sorafenib+immune checkpoint inhibitors; PVTT, portal vein tumor thrombus; type I, tumor thrombi involving segmental branches of portal vein or above; type II, tumor thrombi involving right/left portal vein; type III, tumor thrombi involving the main portal vein.

In patients with type I or II (type I+II) PVTT, the median PFS was 7.2 months (95% CI 2.568–11.832) in the T+S+ICI group and 3.1 months (95% CI 2.708–3.492) in the T+S group (p = 0.031) (**Figure 6A**); the median OS was 12.3 months (95% CI: 10.457–14.143) in the T+S+ICI group and 6.2 months (95% CI 5.416–6.984) in the T+S group (p = 0.076) (**Figure 6B**). In patients with type III PVTT, the median PFS was 6.9 months (95% CI 000–14.028) in the T+S+ICI group and 2.8 months (95% CI 0.967–4.633) in the T+S group (p = 0.001) (**Figure 6C**); the median OS was 10.2 months (95% CI 7.248–13.152) in the T+S+ICI group and 5 months (95% CI 1.835–8.165) in the T+S group (p = 0.004) (**Figure 6D**).

### Univariate and multivariate analyses

In the matched cohort, after screening, ECOG PS and treatments influencing the PFS were selected for multivariate analysis (**Table 3**). The Cox proportional hazard model showed that the ECOG PS (0 + 1 vs. 2) [hazard ratio (HR) = 0.276; 95% CI 0.095–0.800; p = 0.018] and treatment (T+S+ICIs vs. T+S) (HR = 0.376; 95% CI 0.207–0.682; p = 0.001) were independent predictive factors for PFS (**Table 3**). Multivariate analysis indicated that age (<50 vs. ≥50 years) (HR = 2.052; 95% CI 1.040–4.048; p = 0.038) and treatment (T+S+ICIs vs. T+S) (HR = 0.386; 95% CI 0.195–0.764; p = 0.006) were independent predictive factors for OS (**Table 4**).

**Table 3:** Univariate and multivariate predictors of progression-free survival.

Variables	Univariate Cox analysis			Multivariate Cox analysis		
	HR	95% CI	p value	HR	95% CI	p value
Sex (men/women)	1.718	0.795-3.714	0.169			
Age (years) (<50/≥50)	0.851	0.482-1.499	0.576			
ECOG PS (0 + 1/2)	2.819	0.993-8.003	0.052	0.276	0.095-0.800	0.018
HBV infection (positive/negative)	1.312	0.588-2.927	0.507			
Cirrhosis (yes/no)	1.113	0.669-1.723	0.518			
Child–Pugh class (A/B)	0.849	0.459-1.569	0.601			
AFP (ng/mL)(<400/≥400)	0.812	0.457-1.441	0.476			
Tumor size (cm) (<10/≥10)	0.797	0.456-1.394	0.427			
Extrahepatic metastasis (yes/no)	1.176	0.623-1.876	0.685			
PVTT (I+II/III)	0.741	0.517-1.564	0.522			
Albumin level (g/L) (<35/≥35) TBIL (μmol/L) (<20/≥20) ALT (U/L) (<35/≥35) AST (U/L) (<40/≥40) Number of TACE (1/2+3)	0.880 1.290 0.805 1.350 0.829	0.501-1.547 0.726-2.291 0.460-1.408 0.608-2.801 0.467-1.470	0.657 0.385 0.447 0.495 0.521			
Treatment (T+S+ICIs/T+S)	2.483	1.378-4.473	0.002	0.376	0.207-0.682	0.001

ECOG PS, Eastern Cooperative Oncology Group performance status; AFP, alpha-fetoprotein; PVTT, portal vein tumor thrombus; type I, tumor thrombi involving segmental branches of portal vein or above; type II, tumor thrombi involving right/left portal vein; type III, tumor thrombi involving the main portal vein; TBIL, total bilirubin; ALT, alanine transaminase; AST, aspartate transaminase; T+S, transarterial chemoembolization+sorafenib; T+S+ICIs, transarterial chemoembolization+sorafenib+immune checkpoint inhibitors.

**Table 4:** Univariate and multivariate predictors of overall survival.

Variables	Univariate Cox analysis			Multivariate Cox analysis		
	HR	95% CI	p value	HR	95% CI	p value
Sex (men/women)	1.638	0.712-3.768	0.246			
Age (years) (<50/≥50)	0.620	0.325-1.183	0.147	2.052	1.040-4.048	0.038
ECOG PS (0 + 1/2)	2.328	0.811-6.683	0.116	0.473	0.159-1.413	0.180
HBV infection (negative/positive)	1.111	0.433-2.852	0.827			
Cirrhosis (yes/no)	1.211	0.687-1.821	0.649			
Child–Pugh class (A/B)	0.778	0.383-1.577	0.486			
AFP (ng/mL) (<400/≥400)	1.344	0.687-2.631	0.388			
Tumor size (cm) (<10/≥10)	0.786	0.415-1.489	0.461			
Extrahepatic metastasis (yes/no)	1.298	0.795-2.157	0.298			
PVTT (type I+II/III)	0.456	0.452-1.461	0.736			
Albumin level (g/L) (<35/≥35) TBIL (μmol/L) (<20/≥20) ALT (U/L) (<35/≥35) AST (U/L) (<40/≥40) Number of TACE (1/2+3)	1.135 1.083 0.636 1.271 0.590	0.594-2.167 0.554-2.116 0.333-1.217 0.530-3.050 0.307-1.133	0.702 0.816 0.172 0.592 0.113	1.609	0.807-3.208	0.176
Treatment (T+S+ICIs/T+S)	0.426	0.222-0.820	0.011	0.386	0.195-0.764	0.006

ECOG PS, Eastern Cooperative Oncology Group performance status; AFP, alpha-fetoprotein; PVTT, portal vein tumor thrombus; type I, tumor thrombi involving segmental branches of portal vein or above; type II, tumor thrombi involving right/left portal vein; type III, tumor thrombi involving the main portal vein; TBIL, total bilirubin; ALT, alanine transaminase; AST, aspartate transaminase; T+S, transarterial chemoembolization+sorafenib; T+S+ICIs, transarterial chemoembolization+sorafenib+immune checkpoint inhibitors.

### Safety

To assess the safety of the two groups in real clinical practice, the incidence of AEs was reported in cohorts matched previously (**Table 5**); SAEs (more than grade 4) did not occur in this study. Ten (30.3%) patients experienced reactive cutaneous capillary endothelial proliferation (RCCEP) (grade 1/2) on the skin and three (9.2%) patients experienced hypothyroidism (grade 1/2) in the T+S+ICI group; no patient experienced that symptom in the T+S group (respectively, p < 0.05) (**Table 5**). Also, no treatment-related deaths occurred in this study.

**Table 5:** Treatment-related adverse events (TRAE).

Event, n (%)	T+S (n=43)			T+S+ICIs (n=33)			p value		
	Any grade	Grade 1/2	Grade 3/4	Any grade	Grade 1/2	Grade 3/4	Any grade	Grade 1/2	Grade 3/4
Any TRAE	40(93.0)	37(86.0)	6(14.0)	33(100.0)	29(87.9)	7(21.2)	0.122	0.815	0.405
Fatigue	14(32.6)	10(23.3)	4(9.3)	15(45.5)	10(30.3)	5(15.2)	0.251	0.489	0.434
Decreased appetite	12(27.9)	9(20.9)	3(7.0)	15(45.5)	11(33.3)	4(12.1)	0.113	0.224	0.442
Vomiting or nausea	14(32.6)	12(27.9)	2(4.7)	11(33.3)	9(27.3)	2(6.1)	0.943	0.951	0.785
Abdominal pain	12(27.9)	11(25.6)	1(2.3)	10(30.3)	9(27.3)	1(3.0)	0.819	0.868	0.849
Fever	13(30.2)	10(23.3)	3(7.0)	13(39.4)	11(33.3)	2(6.1)	0.404	0.330	0.873
Dose reduce or interruptions	5(11.6)	4(9.3)	1(2.3)	7(21.2)	4(21.1)	3(9.1)	0.256	0.691	0.190
Hypertension	3(7.0)	2(4.7)	1(2.3)	5(15.2)	3(9.1)	2(6.1)	0.250	0.439	0.407
Hand and foot syndrome	8(18.6)	6(14.0)	2(4.7)	8(24.2)	5(15.2)	3(9.1)	0.550	0.883	0.439
Diarrhea	2(4.7)	2(4.7)	0(0.0)	4(12.1)	4(12.1)	0(0.0)	0.231	0.231	_
Alopecia	3(7.0)	3(7.0)	0(0.0)	2(6.1)	2(6.1)	0(0.0)	0.873	0.873	_
Pruritus	5(11.6)	5(11.6)	0(0.0)	6(18.2)	5(15.2)	1(3.0)	0.421	0.652	0.251
Rash	1(2.3)	1(2.3)	0(0.0)	4(12.1)	3(9.1)	1(3.0)	0.088	0.190	0.251
Proteinuria Increased AST	8(18.6) 7(16.3)	6(14.0) 6(14.0)	2(4.7) 1(2.3)	12(36.4) 8(24.2)	9(27.3) 6(18.2)	3(9.1) 2(6.1)	0.081 0.387	0.148 0.616	0.439 0.407
Increased ALT	6(14.0)	4(9.3)	2(4.7)	8(24.2)	6(18.2)	2(6.1)	0.251	0.256	0.785
Decreased neutrophil count	6(14.0)	6(14.0)	0(0.0)	6(18.2)	6(18.2)	0(0.0)	0.616	0.616	–
Increased blood bilirubin	6(14.0)	6(14.0)	0(0.0)	8(24.2)	7(21.2)	1(3.0)	0.251	0.405	0.251
Gastrointestinal hemorrhage	3(7.0)	3(7.0)	0(0.0)	2(6.1)	2(6.1)	0(0.0)	0.873	0.873	–
Hypothyroidism	0(0.0)	0(0.0)	0(0.0)	3(9.1)	3(9.1)	0(0.0)	0.044	0.044	–
RCCEP	0(0.0)	0(0.0)	0(0.0)	10(30.3)	10(30.3)	0(0.0)	<0.001	<0.001	–

Data are numbers of patients, with percentages in parentheses. TACE, transarterial chemoembolization; ICIs, immune checkpoint inhibitors; AST, aspartate aminotransferase; ALT, alanine transaminase; RCCEP, reactive cutaneous capillary endothelial proliferation.

## Discussion

This study revealed that T+S+ICIs conferred a significant survival benefit compared with T+S in patients with BCLC stage C HCC who previously received locoregional treatment. This finding was associated with an increase in median OS from 6.3 to 12.3 months, which might be attributed to the higher ORR and DCR and longer PFS in patients receiving T+S+ICIs rather than those treated with T+S. Multivariate analyses also showed that combining ICIs on the basis of TACE plus sorafenib was an independent predictor for prolonged PFS and OS. These results indicated that the TACE combined with sorafenib and ICI regimen might be a superior treatment option in patients with BCLC C stage HCC who previously received locoregional treatment, which might be due to the following reasons: 1) TACE can lead to tumor necrosis after occlusion of feeding arteries and release of tumor antigens, which can be captured by antigen-presenting cells. This can activate tumor-specific immune responses (18), change the cytokine spectrum and the activity level of T cells and immune cell subsets (18), and transfer TME into Th1 dominance to improve the regulatory T-cell level and obtain favorable survival prognosis (19). 2) Sorafenib may counteract the hypoxia-induced angiogenesis after TACE (12, 20), regulate VEGF-mediated immunosuppression within tumors and TME (21, 22), and enhance the immunomodulatory effect by reversing VEGF-mediated immunosuppression and promoting T-cell infiltration into tumors (23, 24). Therefore, the combination of TACE, sorafenib, and ICIs has a synergistic antitumor effect, contributing to improved survival outcomes in patients with advanced HCC.

Patients with advanced HCC who were administered with nivolumab or pembrolizumab as systemic first‐/second‐/third‐/fourth‐line treatment had an ORR of 12% and a median OS of 11 months (25). However, patients with unresectable HCC who received first-line lenvatinib plus pembrolizumab treatment had an ORR of 46% and a median OS of 12.6 months (26). Thus, combination therapy significantly improved the ORR and OS. A previous study suggested that the median PFS and OS in patients with BCLC C stage TACE-refractory HCC who received TACE+sorafenib+ICI treatments were 10.8 and 13.5 months, respectively, which were higher than the results of this study. The reason may be that the patients’ average liver tumor diameter in the previous study was smaller compared to this study (6.1 ± 2.5 vs. 9.6 ± 4.8 cm) (27). In the TRIPLET study (28), HCC patients in BCLC stage C who received hepatic artery infusion chemotherapy (HAIC) combined with apatinib and camrelizumab had an ORR and DCR of 61.54% and 92.3%, respectively. These results were better than the data obtained in this study, and the reason may be that all patients in the TRIPLET study received no previous treatment (in this study, patients with BCLC C stage HCC were previously treated with locoregional therapy). Cai et al. (29) assessed the TACE+lenvatinib+PD-1 inhibitor for patients with advanced HCC and reported an ORR of 56.1%, a DCR of 85.4%, and a PFS of 7.3 months; these results were consistent with this study.

The main PVTT is the independent risk factor for the survival of HCC (30, 31). In this study, subgroup analyses showed that T+S+ICIs provided a better PFS and OS than T+S in the patients with type III PVTT but not in those with type I+II PVTT. The reason might be that TACE exerted its antitumor property mainly by controlling intrahepatic PVTT rather than extrahepatic PVTT (20). Thus, a treatment strategy combining TACE with a more potent systemic therapy was urgently needed for patients with extrahepatic PVTT. Our results revealed the necessity of the additional treatment with ICIs to TACE plus sorafenib for such patients.

In this study, AEs were mild to moderate and could be managed easily. Chemoembolization- and sorafenib-related AEs were similar to those reported in previous studies (5, 8, 32). The incidence rate of RCCEP (30.3%) was lower in the T+S+ICI group than the result in a previous study (67%) (33). After receiving thyroxine, glucocorticoid, and ICI interruption treatments, patients with hypothyroidism recovered within 2 weeks.

There were several limitations in the present study. Firstly, this study was a retrospective analysis, which carries limitations in terms of selection bias and the control of other confounding factors. We implemented the PSM methodology to account for the effect caused by confounding factors. A randomized clinical trial is required to validate the findings from this study. Secondly, this study has a small sample size. Lastly, only patients with BCLC stage C HCC were included in this study. Thus, the findings from this study may not be generalized to other unresectable HCC populations.

In conclusion, compared with TACE combined with sorafenib, TACE combined with sorafenib plus ICIs is a potentially safe and effective treatment regimen for patients with advanced HCC who previously received locoregional treatment.

## Data availability statement

The raw data supporting the conclusions of this article will be made available by the authors, without undue reservation.

## Ethics statement

The study was reviewed and approved by Sichuan Cancer Hospital. Written informed consent was obtained from the individual(s) for the publication of any potentially identifiable images or data included in this article.

## Author contributions

Conception and design: G-HX and X-QH. Collection and assembly of data: X-GY, Y-YS, H-QW, and D-SL. Manuscript writing: all authors. All authors contributed to the article and approved the submitted version.

## Funding

This study was supported by the Wu Jieping Medical Fund (No. 320.6750.2020-10-122), Beijing Medical Award Found (No. YXJL-2020-0972-0424), a Special Research Fund Project of Tumour Interventional (No. 2020S04), Natural Science Foundation of Sichuan (No. 2022NSFSC0837), and Science and Technology Project of Chengdu (No. 2022-YF05-01811-SN).

## Conflict of interest

The authors declare that the research was conducted in the absence of any commercial or financial relationships that could be construed as a potential conflict of interest.

## Publisher’s note

All claims expressed in this article are solely those of the authors and do not necessarily represent those of their affiliated organizations, or those of the publisher, the editors and the reviewers. Any product that may be evaluated in this article, or claim that may be made by its manufacturer, is not guaranteed or endorsed by the publisher.
